# Determinants of induced abortion among women of reproductive age: evidence from the 2013 and 2019 Sierra Leone Demographic and Health Survey

**DOI:** 10.1186/s12905-023-02175-9

**Published:** 2023-02-01

**Authors:** Foday Robert Sesay, Emmanuel Anongeba Anaba, Adom Manu, Ernest Maya, Kwasi Torpey, Richard M. K. Adanu

**Affiliations:** Department of Population, Family and Reproductive Health, School of Public Health, College of Health Sciences, University of Ghana, Accra, Ghana

**Keywords:** Induced abortion, Determinants, Women of reproductive age, Sierra Leone

## Abstract

**Background:**

Worldwide, pregnancy termination due to unintended pregnancy is crucial in maternal health, particularly in settings where abortion laws are restrictive. Presently, there is a paucity of literature on determinants of induced abortion among women of reproductive age in Sierra Leone. The study findings could be used to improve the country's maternal mortality indices and inform health programs and reproductive health policies geared toward tackling induced abortion.

**Methods:**

We analyzed secondary data from the 2013 and 2019 Sierra Leone Demographic and Health Surveys. The surveys were nationally representative, with weighted samples comprising 16,658 (2013) and 15,574 (2019) women of reproductive age. Descriptive statistics, including frequencies and percentages, were computed, while Chi-square and Binomial Logistics Regression were employed to identify correlates of induced abortion.

**Results:**

The results showed that a minority (9%) of the participants had induced abortion in both surveys. Abortion was significantly associated with age, marital status, employment status, education, parity, and frequency of listening to the radio and watching television (*p* < 0.05). For instance, women aged 45–49 years (AOR = 7.91; 95% CI: 5.76–10.87), married women (AOR = 2.52; 95% CI: 1.95–3.26), and working women (AOR = 1.65; 95% CI: 1.45–1.87) had a higher likelihood of induced abortion compared to their counterparts. Moreover, women with primary education (AOR = 1.27; 95% CI:1.11–1.46) and those who watch television once a week (AOR = 1.29; 95% CI: 1.11–1.49) were more likely to terminate a pregnancy. Women with six or more children (AOR = 0.40; 95% CI: 0.31–0.52) were less likely to terminate a pregnancy compared to those with no child.

**Conclusion:**

The study revealed that a minority of the women had induced abortions. The prevalence of induced abortion did not change over time. Induced abortion was influenced by age, marital status, employment status, education, parity, and exposure to mass media. Therefore, policies and programs to reduce unwanted pregnancies should focus on increasing access to modern contraceptives among women of lower socio-economic status.

## Background

Worldwide, pregnancy termination due to unintended pregnancy is a crucial factor in maternal health, particularly in settings where abortion laws are restrictive. Unsafe abortion affects both the individual and society in terms of health and economic implication [1]. Most women resort to induced abortion because they lack a partner's support, are financially unstable, a victim of rape or incest, and have untimely pregnancies [2]. The World Health Organization (WHO) defines unsafe abortion as a process of terminating a pregnancy by someone lacking the required skills or in a setting that does not meet the minimum medical standards or both [3]. Abortion is classified by WHO as safe when it is done with a method recommended by the WHO, that is appropriate for the gestational age of the pregnancy and the person providing or supporting the abortion is trained [4]. Abortion is less safe when either the method or provider criterion is met, but not both, and least safe when they meet neither the provider nor method criterion [4, 5].

The global estimate for abortion annually is 73.3 million, corresponding to a worldwide abortion rate of 39 abortions per 1000 women aged 15–49 years [6]. The global yearly estimate for unsafe abortion is around 25 million, and the majority of them (97%) occur in the developing world [7]. Globally, the proportion of unintended pregnancies ending in abortion increased from 51% in 1994 to 61% in 2019 [8]. Data from 2010 to 2014 indicated that approximately 55% of abortions worldwide were considered safe, 31% less safe, and 14% least safe [9]. About a million women of reproductive age are hospitalized yearly due to unsafe abortion globally [10] and unsafe abortion accounts for approximately thirteen percent of global maternal deaths [11]. In Sub-Saharan Africa, unsafe abortion is estimated to have killed one woman every eight minutes in 2015 [12].

It has been established that countries with restrictive abortion laws have higher maternal mortality [4]. Sierra Leone is among those countries with restrictive abortion laws. According to the Center for Reproductive Rights (2009), the country is classified in category three in the world's abortion law, meaning abortion is performed when it is geared toward saving the mother's life. The country's law on abortion was inherited from the British colonial government. It states that women who attempt to abort are guilty of a crime and " shall be liable and sentenced to life" [13]. The above unfortunate situation, coupled with the fact that women want to avoid the stigma created by religious and cultural influences have caused many women to resort to unsafe abortion practices. It has also caused a dilemma among caregivers to perform their duty as health care providers or obey the law [14]. In December 2015, Sierra Leone attempted to revoke this law (Section 58 of the 1861 offenses against the Person Act) in the Safe Abortion Bill, allowing abortion on request [15]. However, because of concerns raised by religious leaders in the whole of Sierra Leone, this bill was not signed into law.

The quality of health care services in Sierra Leone has been a significant problem coupled with limited access to sexual and reproductive health services, partly due to the eleven years of civil war followed by the Ebola outbreak in 2014. In addition, there is a severe shortage of trained medical personnel to provide the needed health services. For example, in contrast to the WHO recommendation of 23 skilled health providers per 10,000 population, the country has about two qualified skilled health providers (physicians, midwives, and nurses) per 10,000 population [16]. Furthermore, the situation of women having induced abortions is made worse by the low modern contraceptive uptake (24%) among women of reproductive age [17]. Sierra Leone’s maternal mortality ratio of 717 maternal deaths per 100, 000 live births is one of the worst worldwide [18]. Of the direct causes of maternal mortality in Sierra Leone, unsafe abortion is ranked fifth, trending behind obstetric hemorrhage, hypertension, obstructed labor, and sepsis. Moreover, unsafe abortion contributes to about 10% of Sierra Leone's maternal mortality ratio [19]. In addition, a study [2] estimated the cost of treatment and impact of unsafe abortion in Sierra Leone as $35 for simple post-abortion care (PAC) with $166 and $272 for moderate and severe complications, respectively [2].

Induced abortion in Sierra Leone has not been extensively investigated. Previous studies among women of reproductive age have sought to examine why women resort to abortion, especially from unskilled providers, as well as their knowledge and use of contraceptives [2, 20]. In addition, a study on the influence of international and regional human rights treaties on domestic abortion policies in Sierra Leone revealed that women are dying from unsafe abortion [21]. Notwithstanding, there is a paucity of literature on the determinants of induced abortion among women of reproductive age in Sierra Leone. In order to address the obstacles to obtaining safe abortion treatment in Sierra Leone, this study examines factors influencing induced abortion among women.

## Methods

### Study location, design, and data source

Sierra Leone is located on the west coast of Africa and covers an area of 72,000 square kilometers [18]. It shares a border with Guinea on the north and northeast, Liberia on the east and southeast, and the west by the Atlantic Ocean [18]. According to the 2015 Population and Housing Census, the country has a total population of 7,092,113 with just over half being female (50.8%) [24]. This study analyzed the women's data from the two most recent 2013 and 2019 Sierra Leone Demographic and Health Surveys (SLDHS) [18, 22]. The DHS is a household-based, nationally representative survey. It uses a two-stage sample design. For instance, in the 2013 DHS, the first stage involved selecting 435 enumeration areas from 27 strata with probability proportional to size, using the 2004 Population and Housing Census report [23], while the second comprised the selection of 30 households from each cluster. A total number of 13,006 households within the enumeration areas were selected. We obtained 16,658 women as the weighted sample size of women aged 15–49 years.

Similarly, in the 2019 DHS, the first stage comprised the selection of 578 enumeration areas from 31 strata, proportional to size employing the 2015 Population and Housing Census report [24], while the second stage involved the selection of 24 households from each cluster, resulting in a total sample size of approximately 13,872. A total of 15,574 women aged 15–49 years were obtained as a weighted sample. The target population was women of reproductive age who had ever terminated a pregnancy and passed the night before the survey in the selected households.

The anonymized data was cleaned, missing values were dropped and adjusted for the complex nature of the survey. Permission to use the DHS data was sought from Measure DHS. The anonymized datasets were only downloaded on approval of the request to undertake this analysis. The data analysed in this study were saved on a password-protected personal computer. The data was declared survey data using sampling weight, weight, and strata or employing the 'svy' STATA command. Detailed information about the 2013 and 2019 DHS is included elsewhere [18, 22].

### Measurements

The dependent variable in this study was ever terminated a pregnancy (induced abortion), coded as yes = 1 and no = 0. The independent variables mentioned in the literature include those characteristics of the women who attest to having terminated a pregnancy. These include women's age (15–19 = 1; 20–24 = 2; 25–29 = 3; 30–34 = 4; 35–39 = 5; 40–44 = 6; 45–49 = 7), educational status (no education = 1; primary = 2; secondary = 3; higher = 4), employment status (not working = 1; working = 2), wealth index (poorest = 1; second = 2; middle = 3; fourth = 4; richest = 5), religion (Christianity = 1; Muslim = 2; others religion = 3), place of residence (urban = 1; rural = 2), marital status (never in union = 1; married/in union = 2; single (formerly married/in union) = 3), and parity (none = 1; 1–2 children = 2; 3–5 children = 3; 6 or more children = 4). Other independent variables were current contraceptive use (no method = 1; modern method = 2; traditional method = 3), knowledge about ovulation, correct (halfway between two menstrual periods) = 1; incorrect = 2; don’t know = 3), frequency of reading newspaper, listening to radio and watching television (not at all = 1; less than once a week = 2; at least once a week = 3).

### Statistical analyses

All analyses were carried out using STATA/SE version 16 (Stata Corp, College Station., Texas, USA). Descriptive statistics of the background characteristic of respondents were computed and summarized (Table 1). At the bivariate level, the Chi-squared test was used to determine the association between variables under study and the outcome of interest. Similarly, at the multivariable level, binary logistics regression was used to determine the predictors of induced abortion among women of reproductive age. In all, three models were computed. Model 1 looked at predictors of induced abortion in 2013, while model 2 focused on predictors of induced abortion in 2019. The third model (model 3) focused on predictors of induced abortion in 2013 and 2019 (combined) while adjusting for the survey year. The significance for the analysis was set at *p* < 0.05, while the strength of association was examined using odds ratios and their 95% confidence interval.

**Table 1:** Participant characteristics

Characteristic	2013 (N = 16,658) n (%)	2019 (N = 15,574) n (%)	2013–2019 (N = 32,232) n (%)
*Age group*			
15–19	3878(23)	3427 (22)	7305(23)
20–24	2683(16)	2629 (17)	5312(16)
25–29	2843(17)	2728 (18)	5571(17)
30–34	2287(14)	1942 (12)	4229(13)
35–39	2260(14)	2224 (14)	4484(14)
40–44	1362(8)	1337 (9)	2699(8)
45–49	1344(8)	1288 (8)	2632(8)
*Marital status*			
Never in union	4730(28)	5058(32)	9788(30)
Married/in a union	10,903(65)	9715(62)	20,618(64)
Single	1025(6)	801(5)	1826(6)
*Employment status*			
Not working	5319(32)	4831(31)	10,150(31)
Working	11,339(68)	10,743(69)	22,082(69)
*Residence*			
Urban	5933(36)	7163 (46)	13,096(41)
Rural	10,725(64)	8411 (54)	19,136(59)
*Educational status*			
No education	9293(56)	7081(45)	16,375(51)
Primary	2331(14)	2103(14)	4433(14)
Secondary	4533(27)	5724(37)	10,257(32)
Higher	501(3)	666(4)	1167(4)
*Wealth index*			
Poorest	3089(19)	2738(18)	5828(18)
Second	3046(18)	2831(18)	5877(18)
Middle	3140(19)	2954(19)	6093(19)
Fourth	3388(20)	3385(22)	6773(21)
Richest	3994(24)	3666(24)	7660(24)
*Religion*			
Christianity	3527(21)	3616(23)	7143(22)
Muslim	13,032(78)	11,953(77)	24,985(78)
Others	99(1)	6(0)	105(0)
*Parity*			
None	4168(25)	4361(28)	8529(26)
1–2	4459(27)	5224(34)	9683(30)
3–5	5119(31)	4893(31)	10,011(31)
6 or more	2912(17)	1097(7)	4008(12)
*Current contraceptive use*			
No method	12,982(78)	11,794(76)	24,776(77)
Modern method	3361(20)	3696(24)	7058(22)
Traditional method	315(2)	83(1)	398(1)
*Knowledge of ovulation*			
Correct	4882(29)	7867(51)	12,748(40)
Incorrect	6694(40)	5865(38)	12,558(39)
Don’t know	5083(31)	1842(12)	6925(21)
*Frequency of reading newspaper*			
Not at all	14,844(89)	14,320(92)	29,164(90)
Less than once a week	666(4)	851(5)	1517(5)
At least once a week	1149(7)	403(3)	1552(5)
*Frequency of listening to the radio*			
Not at all	6325(38)	8653(56)	14,978(46)
Less than once a week	3674(22)	3182(20)	6856(21)
At least once a week	6659(40)	3739(24)	10,399(32)
*Frequency of watching television*			
Not at all	13,217(79)	11,143(72)	24,359(76)
Less than once a week	1090(7)	2109(14)	3199(10)
At least once a week	2351(14)	2322(15)	4674(15)
*Ever had a pregnancy terminated*			
No	15,099(91)	14,246(91)	29,345(91)
Yes	1559(9)	1328(9)	2887(9)

## Results

### Descriptive statistics of participant characteristics

The study analyzed data from 16,658 women and 15,1574 women in the 2013 and 2019 SLDHS respectively. In both surveys, the prevalence of induced abortion was 9%. In the 2013 survey, 36% of the participants resided in urban areas compared to 46% in 2019 survey. In addition, a higher proportion of the participants in the 2019 survey (37%) had secondary education compared to the 2013 survey (27%). The use of modern contraceptives had increased from 20% in 2013 to 24% in 2019. Similarly, accurate knowledge about ovulation had increased from 29% in 2013 to 51% in 2019 (Table 1).

### Association between participant characteristics and termination of pregnancy

In both 2013 and 2019, induced abortion was significantly associated with age, marital status, employment status, education and parity, (*p* < 0.05). In 2019, knowledge about ovulation, frequency of listening to the radio, reading newspapers were significantly associated with abortion (*p* < 0.05). In the combined analysis, induced abortion was associated with age, marital status, employment status, educational status, parity, frequency of reading newspaper and frequency of listening to radio (*p* > 0.05) (Table 2).

**Table 2:** Cross-tabulation of participant characteristics and abortion among women of reproductive age in Sierra Leone

Characteristic	Ever had a pregnancy terminated								
	2013 SLDHS			2019 SLDHS			2013–2019 SLDHS		
	No	Yes	Χ^2^	No	Yes	Χ^2^	No	Yes	Χ^2^
*Age group*									
15–19	3786(98)	92(2)	48.06*	3386(99)	40(1)	56.86 *	7172(98)	133(2)	102.43 *
20–24	2501(93)	182(7)		2486(95)	143(5)		4986(94)	325(6)	
25–29	2523(89)	320(11)		2476(91)	252(9)		4999(90)	572(10)	
30–34	2022(88)	265(12)		1715(88)	227(12)		3737(88)	492(12)	
35–39	1944(86)	316(14)		1936(87)	288(13)		3880(87)	604(13)	
40–44	1170(86)	193(14)		1154(86)	183(14)		2323(86)	376(14)	
45–49	1155(86)	190(14)		1093(85)	195(15)		2247(85)	385(15)	
*Marital status*									
Never in union	4574(97)	157(3)	81.02*	4932(98)	126(2)	86.46*	9506(97)	282(3)	167.74 *
Married/in union	9644(88)	1258(12)		8619(89)	1096(11)		18,263(89)	2355(11)	
Single	881(86)	144(14)		695(87)	106(13)		1577(86)	250(14)	
*Employment status*									
Not working	5054(95)	265(5)	118.15 *	4633(96)	198(4)	120.59*	9687(95)	463(5)	236.58 *
Working	10,045(89)	1294(11)		9613(89)	1130(11)		19,658(89)	2424(11)	
*Residence*									
Urban	5388(91)	545(9)	0.08	6589(92)	575(8)	1.44	11,977(91)	1120(9)	1.19
Rural	9711(91)	1014(9)		7657(91)	753(9)		17,369(91)	1767(9)	
*Educational status*									
No education	8289(89)	1004(11)	17.95*	6342(90)	740(10)	27.59*	14,631(89)	1744(11)	44.92 *
Primary	2114(91)	217(9)		1897(90)	206(10)		4011(90)	423(10)	
Secondary	4260(94)	273(6)		5428(95)	296(5)		9688(94)	569(6)	
Higher	436(87)	65(13)		580(87)	86(13)		1016(87)	151(13)	
*Wealth index*									
Poorest	2832(92)	258(8)	0.83	2462(90)	276(10)	1.55	5294(91)	534(9)	0.30
Poorer	2739(90)	307(10)		2585(91)	246(9)		5324(91)	553(9)	
Middle	2832(90)	308(10)		2729(92)	225(8)		5561(91)	533(9)	
Richer	3089(91)	299(9)		3095(91)	290(9)		6184(91)	589(9)	
Richest	3608(90)	387(10)		3375(92)	291(8)		6983(91)	678(9)	
*Religion*									
Christianity	3239(92)	288(8)	2.56	3302(91)	314(9)	0.08	6541(92)	601(8)	1.10
Muslim	11,769(90)	1264(10)		10,939(92)	1014(8)		22,708(91)	2277(9)	
Others	91(93)	7(7)		5(88)	1(12)		96(92)	8(8)	
*Parity*									
None	3992(96)	176(4)	47.94 *	4165(96)	196(4)	30.75*	8157(96)	372(4)	75.58 *
1–2	4076(91)	383(9)		4732(91)	491(9)		8808(91)	875(9)	
3–5	4499(88)	619(12)		4362(89)	531(11)		8861(89)	1150(11)	
6 or more	2532(87)	380(13)		987(90)	110(10)		3519(88)	489(12)	
*Current contraceptive use*									
No method	11,738(90)	1243(10)	1.17	10,779(91)	1015(9)	0.92	22,518(91)	2258(9)	
Modern method	3070(91)	291(9)		3394(92)	303(8)		6464(92)	594(8)	
Traditional method	291(92)	24(8)		73(88)	10(12)		364(91)	35(9)	
*Knowledge of ovulation*									
Correct	4403(90)	479(10)	0.47	5337(91)	681(9)	3.07*	11,589(91)	1160(9)	0.46
Incorrect	6086(91)	608(9)		7186(91)	527(9)		11,423(91)	1135(9)	
Don’t know	4611(91)	472(9)		1722(93)	120(7)		6333(91)	592(9)	
*Frequency of reading newspaper*									
Not at all	13,422(90)	1422(10)	2.91	13,096(91)	1224(9)	9.72*	26,518(91)	2646(9)	3.03 *
Less than once a week	610(92)	56(8)		805(95)	46(5)		1415(93)	102(7)	
At least once a week	1068(93)	81(7)		344(85)	59(15)		1412(91)	139(9)	
*Frequency of listening to a radio*									
Not at all	5791(92)	534(8)	2.34	8020(93)	633(7)	8.05*	13,810(92)	1168(8)	10.65 *
Less than once a week	3308(90)	365(10)		2865(90)	317(10)		6173(90)	682(10)	
At least once a week	6000(90)	659(10)		3361(90)	378(10)		9361(90)	1037(10)	
*Frequency of watching television*									
Not at all	11,959(90)	1258(10)	0.61	10,208(92)	935(8)	1.32	22,167(91)	2192(9)	1.03
Less than once a week	999(92)	91(8)		1944(92)	165(8)		2943(92)	256(8)	
At least once a week	2141(91)	210(9)		2094(90)	229(10)		4235(91)	439(9)	

^*^p-value < 0.05; *SLDHS* Sierra Leone demographic and health survey, *X*^*2*^ Chi-square

### Predictors of termination of pregnancy among women of reproductive age in Sierra Leone

In the adjusted analysis for model 1, we found that the respondent's age, marital status, employment status, parity, and exposure to radio were significant predictors of induced abortion in the 2013 SLDHS (*p* < 0.05). For example, women aged 45–49 years (AOR = 4.60; 95%CI: 3.05–6.94) were about four times more likely to terminate a pregnancy compared to those aged 15–19. Also, women who were employed (AOR = 1.71; 95% CI: 1.45–2.02) were about twice more likely to terminate a pregnancy compared to those who were unemployed. In the adjusted analysis for model 2, respondent age, marital status, employment status, education, parity, and frequency of listening to the radio and reading newspapers were significant predictors of induced abortion. For example, women who listen to the radio (AOR = 1.57; 95% CI: 1.23–2.01) had high odds of terminating a pregnancy compared with those who do not listen to the radio. Also, women who had primary education (AOR = 1.37; 95% CI: 1.12–1.69) were more like to terminate a pregnancy compared to those with no education. In the adjusted analysis for model 3, the significant predictors of induced abortion were age, marital status, employment status, education, parity, and exposure to the radio. For instance, women aged 45–49 years (AOR = 7.91; 95% CI: 5.76–10.87), married women (AOR = 2.52; 95% CI: 1.95–3.26), working women (AOR = 1.65; 95% CI: 1.45–1.87) had a higher likelihood of terminating a pregnancy compared with their counterparts (Table 3).

**Table 3:** Logistic regression analysis of predictors of abortion among women of reproductive age in Sierra Leone

	Model 1 (2013 SLDHS)	Model 2 (2019 SLDHS)	Model 3 (2013–2019 SLDHS)
Characteristic	AOR 95% CI	AOR 95% CI	AOR 95% CI
*Age group*			
15–19	1(ref)	1(ref)	1(ref)
20–24	2.39(1.65–3.47)*	3.97(2.51–6.26)*	2.91(2.19–3.87)*
25–29	3.74(2.56–5.46)*	6.58(4.01–10.78)*	4.74(3.58–6.36)*
30–34	3.72(2.52–5.49)*	9.64(5.87–15.83)*	5.68(4.22–7.66)*
35–39	4.67(3.11–6.99)*	11.37(6.96–18.56)*	6.98(5.15–9.45)*
40–44	4.71(3.09–7.17)*	13.05(7.98–21.35)*	7.45(5.44–10.21)*
45–49	4.60(3.05–6.94)*	15.06(9.04–25.08)*	7.91(5.76–10.87)*
*Marital status*			
Never in union	1(ref)	1(ref)	1(ref)
Married/in union	2.09(1.48–2.95)*	3.00(2.03–4.42)*	2.52(1.95–3.26)*
Single	2.25(1.51–3.35)*	2.84(1.69–4.76)*	2.59(1.88–3.55)*
*Employment status*			
Not working	1(ref)	1(ref)	1(ref)
Working	1.71(1.45–2.02)*	1.54(1.26–1.88) *	1.65(1.45–1.87)*
*Residence*			
Urban	1(ref)	1(ref)	1(ref)
Rural	0.99(0.73–1.35)	0.97(0.76–1.24)	0.97(0.79–1.19)
*Educational status*			
No education	1(ref)	1(ref)	1(ref)
Primary	1.19(0.98–1.44)	1.37(1.12–1.69)*	1.27(1.11–1.46)*
Secondary	1.16(0.91–1.47)	0.98(0.74–1.29)	1.04(0.86–1.24)
Higher	1.62(0.93–2.83)	1.16(0.81–1.66)	1.34(0.98–1.84)
*Wealth index*			
Poorest	1(ref)	1(ref)	1(ref)
Second	1.19(0.93–1.53)	0.87(0.69–1.09)	1.02(0.86–1.20)
Middle	1.18(0.92–1.52)	0.81(0.63–1.03)	0.98(0.82–1.17)
Fourth	1.15(0.84–1.57)	0.99(0.73–1.34)	1.06(0.85–1.33)
Richest	1.46(0.94–2.26)	0.76(0.51–1.14)	1.06(0.78–1.44)
*Religion*			
Christianity	1(ref)	1(ref)	1(ref)
Muslim	1.13(0.92–1.38)	1.02(0.85–1.22)	1.06(0.93–1.21)
Others	0.92(0.39–2.15)	1.73(0.18–16.20)	0.92(0.41–2.03)
*Parity*			
None	1(ref)	1(ref)	1(ref)
1–2	0.73(0.53–1.01)	0.47(0.36–0.62)*	0.59(0.47–0.72)*
3–5	0.70(0.51–0.97)*	0.30(0.23–0.40)*	0.45(0.37–0.56)*
6 or more	0.69(0.47–1.01)*	0.23(0.17–0.33)*	0.40(0.31–0.52)*
*Current contraceptive use*			
No method	1(ref)	1(ref)	1(ref)
Modern method	0.97(0.79–1.19)	1.08(0.91–1.29)	1.01(0.89–1.16)
Traditional method	0.76(0.46–1.26)	1.43(0.69–2.94)	0.91(0.60–1.38)
*Knowledge of ovulation*			
Correct	1(ref)	1(ref)	1(ref)
Incorrect	0.93(0.76–1.14)	0.99(0.84–1.16)	0.96(0.85–1.10)
Don’t know	1.04(0.86–1.26)	0.04(0.78–1.39)	1.06(0.91–1.24)
*Frequency of reading newspaper*			
Not at all	1(ref)	1(ref)	1(ref)
Less than once a week	1.05(0.72–1.52)	0.67(0.45–0.99) *	0.85(0.64–1.12)
At least once a week	0.74(0.49–1.13)	1.72(1.11–2.65) *	1.00(0.74–1.35)
*Frequency of listening to a radio*			
Not at all	1(ref)	1(ref)	1(ref)
Less than once a week	1.27(1.01–1.60) *	1.57(1.23–2.01) *	1.39(1.18–1.64) *
At least once a week	1.27(1.04–1.54) *	1.24(0.97–1.59)	1.29(1.11–1.49) *
*Frequency of watching television*			
Not at all	1(ref)	1(ref)	1(ref)
Less than once a week	0.85(0.63–1.15)	0.89(0.69–1.15)	0.87(0.72–1.06)
At least once a week	0.97(0.74–1.28)	1.30(0.96–1.77)	1.10(0.90–1.35)
*Year of survey*			
2013			1(ref)
2019			0.89(0.76–1.04)

^*^p-value < 0.05, *SLDHS* Sierra Leone demographic and health survey, *AOR* Adjusted odd ratio, *CI* Confidence interval

## Discussion

The prevalence of women who ever had a pregnancy terminated was 9% in both the 2013 and 2019 SLDHS, which is consistent with studies reported in Mozambique [25] and Ethiopia [26] but lower (25%) than a study done in Ghana [25]. The reason for the difference between Sierra Leone and Ghana might be the differences in the study period, target population, and the increased access to maternal health care services over the years. However, the prevalence of induced abortion in our study was found to be higher than in a study done among female university students in Wolaiytasodo, Ethiopia [27]. A possible explanation might be the difference in the study population. We utilized national-level data based on SLDHS, while the study in Wolaiytasodo Ethiopia was conducted among a particular population (female university students).

Our study found a statistically significant relationship between pregnancy termination and age, with the odds higher among women 45–49 years. This finding is congruent with prior studies conducted in Ethiopia [27], Ghana [25], and Mozambique [25], where older women experienced more abortion occurrences compared to their younger counterparts. This could be partly explained by the fact that older women are predisposed to medical and pregnancy-related complications like cardiovascular disease, diabetes mellitus and chromosomal abnormality, which could complicate the pregnancy and result in a poor prognostic outcome [28]. Similarly, they may have attained their desired family size. On the contrary, a study in Ethiopia [29] reported that ever having a pregnancy terminated was higher in youth and young adults than in older women.

The current study showed that maternal education was a significant predictor of induced abortion. Women with primary education were more likely to have a terminated pregnancy than uneducated women. This relationship is consistent with the report from a study done in Ethiopia [27]. Educated women are more likely to afford abortion services or more knowledgeable about abortion service providers and laws [30].

It was also observed that the odds of terminating pregnancy were higher among working women than women who were not employed. This is consistent with previous studies done in Mozambique [25] and Ghana [25]. The high prevalence of pregnancy termination among employed women can be partly explained by the fact that they are financially empowered and can afford the cost involved in terminating a pregnancy compared to their unemployed counterparts. In addition, it might be due to the increase in knowledge and self-responsibilities as a working woman.

In this study, media exposure was a significant predictor associated with increased odds of induced abortion. These findings concur with studies from Ethiopia [26], Ghana [25], and Mozambique [25]. It could be due to the reason that the media serve as an important channel of providing information about abortion care. Furthermore, women who have access to mass media may be knowledgeable about abortion laws and abortion pills [31, 32].

Regarding parity, the current study found that women with parity of six and above were less likely to terminate a pregnancy than women with no children. This finding confirms what was found in a study done in Ghana [25] and Mozambique [25]. These studies reported that women with no children were more likely to terminate a pregnancy than those with parity four and above. It was argued that women with no children are most likely to be adolescents. They face challenges of unmet family planning and unintended pregnancies.

It was observed that the prevalence of induced abortion was low among unmarried women compared to women with other marital statuses. This corroborates the findings reported in previous studies done in Ethiopia [33] and Nigeria [34]. Contrary to our findings, a study in Nepal [35] explained that the high prevalence of abortion among unmarried women is expected due to the undesirable attitude of medical personnel, society, and family members towards never-married women. Similarly, the current study also found that pregnancy termination was high among single women. A possible explanation might be that these women are without husbands, hence they are more likely to be single parent. Besides, there is stigma associated with having children out of wedlock in most Africa countries.

### Implications of the findings in this study

The findings from this study have implications for abortion policy, programming and research. Induced abortion constitutes a health problem among women of reproductive age. Therefore, the relevant authorities must provide comprehensive and culturally appropriate sexual and reproductive health services for women. Programs addressing women's education and livelihood should be set up to help them make informed choices like contraceptive use and prevention of unwanted pregnancies. Presently, there is a paucity of literature in Sierra Leone on the sociodemographic correlates of induced abortion among women of reproductive age. This study set the platform for future research on the subject matter to aid policymakers and programmers in decision-making and program planning.

### Strengths and limitations of the study

A major strength of this study is that the analysis used nationally representative data, following international standards in every country. This study is the first study in Sierra Leone to assess the sociodemographic determinants of induced abortion. These findings should be interpreted with caution because cross-sectional studies cannot confirm causal relationships. Also, since abortion is a culturally sensitive issue and is based on self-reporting, there may be the possibility of social desirability bias that led to under-reporting.

## Conclusion

This study revealed that a minority of Sierra Leonean women of reproductive age had ever terminated a pregnancy. Older age, higher education, being employed, exposure to mass media, being single, and low parity were significant determinants of induced abortion. Our study findings provide relevant information for maternal health policy and planning. We recommend that interventions aimed at reducing induced abortion should focus on reducing unwanted pregnancies through increasing access to modern contraceptives among women of low socio-economic status.

## Data Availability

All data generated or analysed during this study are included in this published article [and its supplementary information files].

## Abbreviations

CI: Confidence interval
DHS: Demographic health survey
MMR: Maternal mortality ratio
TOP: Termination of pregnancy
SA: Spontaneous abortion
SLDHS: Sierra Leone Demographic and Health Survey
WHO: World Health Organization
